# Vestibular contribution to path integration deficits in ‘at-genetic-risk’ for Alzheimer’s disease

**DOI:** 10.1371/journal.pone.0278239

**Published:** 2023-01-03

**Authors:** Gillian Coughlan, William Plumb, Peter Zhukovsky, Min Hane Aung, Michael Hornberger

**Affiliations:** 1 Department of Neurology, Massachusetts General Hospital, Harvard Medical School, Boston, MA, United States of America; 2 Department of Computing, Imperial College London, London, United Kingdom; 3 Centre for Addiction and Mental Health, Kimel Family Translational Imaging Genetics Laboratory, Toronto, Ontario, Canada; 4 School of Computing Sciences, University of East Anglia, Norwich, United Kingdom; 5 Norwich Medical School, University of East Anglia, Norwich, United Kingdom; Cardiff University, UNITED KINGDOM

## Abstract

Path integration changes may precede a clinical presentation of Alzheimer’s disease by several years. Studies to date have focused on how spatial cell changes affect path integration in preclinical AD. However, vestibular input is also critical for intact path integration. Here, we developed the vestibular rotation task that requires individuals to manually point an iPad device in the direction of their starting point following rotational movement, without any visual cues. Vestibular features were derived from the sensor data using feature selection. Machine learning models illustrate that the vestibular features accurately classified *Apolipoprotein E* ε3ε4 carriers and ε3ε3 carrier controls (mean age 62.7 years), with 65% to 79% accuracy depending on task trial. All machine learning models produced a similar classification accuracy. Our results demonstrate the cross-sectional role of the vestibular system in Alzheimer’s disease risk carriers. Future investigations should examine if vestibular functions explain individual phenotypic heterogeneity in path integration among Alzheimer’s disease risk carriers.

## Introduction

Alzheimer’s disease (AD) is a common neurodegenerative condition afflicting one’s ability to update self-motion information. This process, known as path integration, is partially mediated by spatial cells in the entorhinal cortex and hippocampus [1], both of which are altered by AD pathophysiology in the preclinical stage [2–4]. A major challenge at this stage, however, is to understand the mechanisms underlying path integration impairment in preclinical AD. Understanding modifiable factors that influence variability in path integration will help pinpoint pre-disease pathological changes and in turn guide preclinical treatment targets.

Beyond demographic factors such as age and sex [5], vestibular function is a predictor of declining PI in animal models, with substantial evidence that the vestibular system transmits specific types of information about self-motion to structures such as the hippocampus and entorhinal cortex [6–11]. Retrospective epidemiological studies in humans also show that vestibular dysfunction originating from the otolith and semicircular canals is associated with cognitive deficits in the elderly, including spatial deficits [12, 13]. Length of time with vestibular dysfunction may contribute to impairment severity [14]. Crucially, experimental studies in humans show that vestibular signals provide robust signals for path integration, suggesting that vestibular signals may contribute to impaired self-motion perception in preclinical AD, particularly given epidemiological research showing that vestibular dysfunction is three times more common in AD patients compared to age-matched controls [15, 16].

Vestibular dysfunction and its precipitating factors (such as hearing loss; see recent Review Smith, 2021, vestibular migrane and vertigo) are at least partially modifiable [14, 17, 18]. Thus, identifying and treating early vestibular dysfunction may serve a preventative role in AD. Building on the work of Mittelstaedt and colleagues [20], we developed a behavioural vestibular paradigm that is based on hand movements. Given the high-dimensional nature of this movement data, we applied machine learning algorithms to analyse patterns of movement, which we term ‘vestibular features’. The task was designed to test both path integration and vestibular function in mid-life adults.

The task was administered to apolipoprotein (*APOE*) ε4 allele carriers, of whom almost half develop Alzheimer’s disease by the average age of 76, compared to only 20% of non-carriers who convert to Alzheimer’s disease by the average age of 84 [19]. Using machine learning, we aimed to (i) determine whether vestibular features classify ε4 carriers from non-carrier controls with and without the inclusion of demographic information and (ii) determine whether vestibular features classify ε4 carriers from non-carrier controls with and without the inclusion of PI accuracy. We hypothesised that each machine learning model would distinguish at-genetic risk *APOE* ε4 carriers from non ε4 carriers. Given the subtle nature of cognitive deficits in mid-life adults, we expected that classification accuracy would increase with each consecutive task trial. As machine learning techniques are not commonly applied to cognitive data in preclinical AD research, we applied three machine learning algorithms to explore if each algorithm consistently produced a similar classification accuracy.

## Materials and methods

### Participants and procedure

One hundred and fifty participants between 50 and 75 years of age were recruited to participate in a research study at the University of East Anglia, Norwich, UK. Written consent was obtained from all participants and ethical approval was obtained from Faculty of Medicine and Health Sciences Ethics Committee at the University of East Anglia, Reference FMH/2016/2017–11. Participants with a history of psychiatric or neurological disease, substance use disorder or motor control disorder were excluded. Participants receiving anti-depression or anti-anxiety medication at the time of screening were excluded. Saliva kits were sent to participants’ home and returned to the university on the same day the saliva sample was taken to determine *APOE* genotype status. Sensor data were collected on the iPad-based assessment tool (see *The Vestibular Rotation Task* for details), These data were only collected during the follow-up visit of the study, 18-months after the baseline assessments that are published. As just 25% of the population carry an *APOE* ε4 allele (23% *APOE* ε3ε4, 2% *APOE* ε4ε4 [19], 31 ε3ε4 carriers were detected. We selected a subset of ε3ε3 carriers that form the majority of the population (75%) to match the ε3ε4 group for age and sex. Ten ε3ε4 did not complete task at the follow-up timepoint due to study attrition and technological problems, leaving the final sample size at 53 participants (32 ε3ε3 carriers and 21 ε3ε4 carriers). We did not include a third genetic subgroup of homozygous *APOE* ε4 carriers, because they were too rare (*n* = 3), although their scores are reported in S1 File. *APOE* ε2 carriers (15% of the UK population) were also excluded as it is unclear how the ε2 allele acts on cognitive performance or the further development of AD. The sample size required for the present study was determined based on similar studies [20–23].

### *APOE* genotyping

A saliva sample was collected for direct genotyping of *APOE*. See [24] for further details.

### Vestibular rotation task

**Fig 1:**
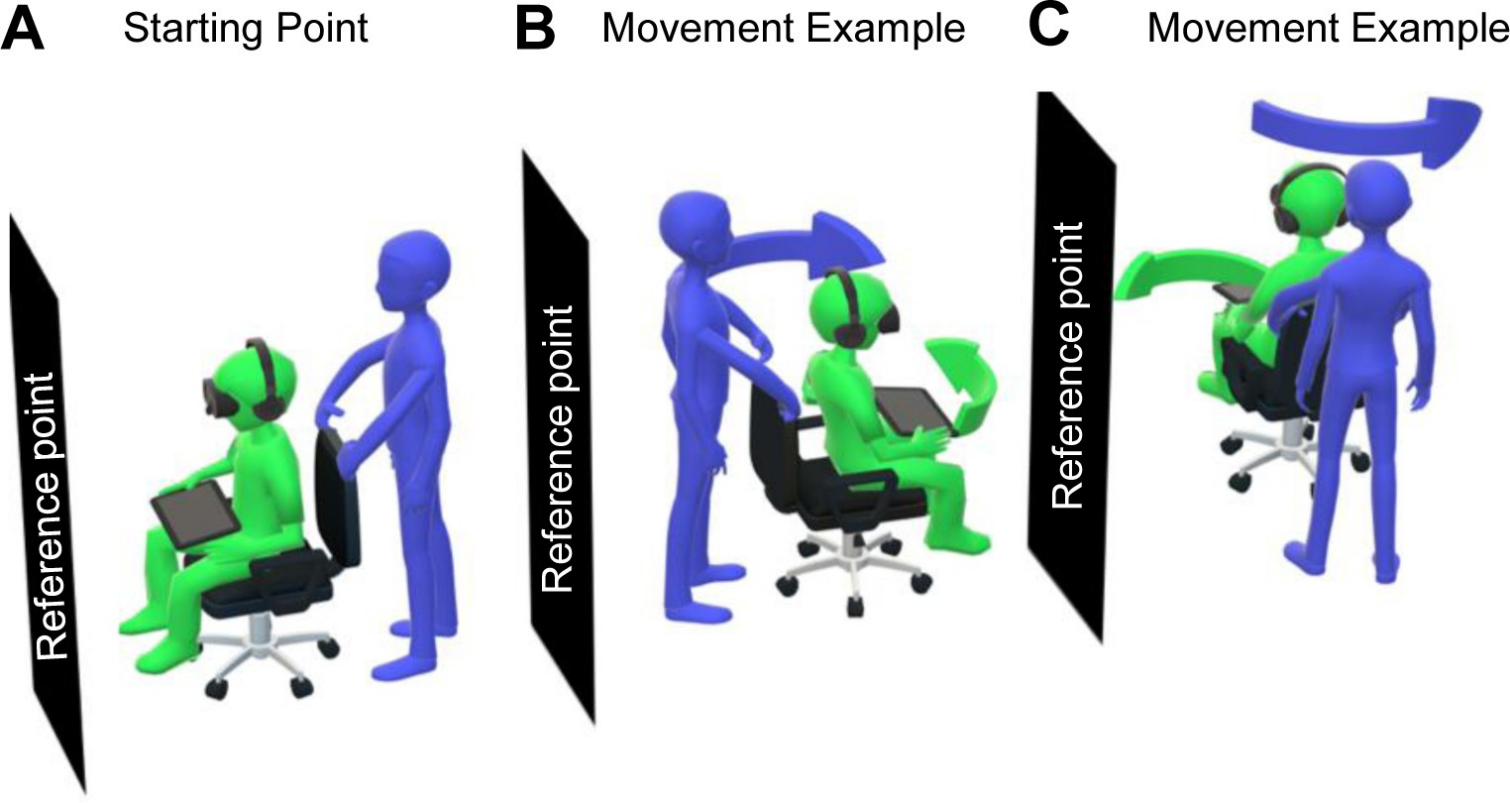
Semantic representation of the vestibular rotation task.

To isolate the input of vestibular signals to performance, the task was administered in the complete absence of external visual or auditory cues. Given the subtle nature of cognitive deficits in mid-life adults, the complexity of the task increased in each consecutive task trial (rotations are detailed in Table 1). During the task: 1) Participants sat in the rotating chair (feet not touching the floor) and held an iPad flat in their hands (Fig 1A). 2) The participant was blindfolded and given earplugs on to ensure the test tapped into the vestibular system with no external stimuli. 3) Participants were told to remember the object in front of them (i.e. door) as their reference point. 4) The x, y, z co-ordinates of the reference point were recorded on the iPad. 5) During the trials, the examiner rotated the participant in the chair (see purple arrows in Fig 1B and 1C) and always remained behind them to avoid serving as a location cue. 6) Three seconds following the turn completion, participants were asked to point the iPad as accurately as possible in the direction of the reference point, while still wearing their blindfold and earplugs (see green arrows in Fig 1B and 1C). 7) During the pointing back movement response, the iPad sensors recorded accelerometer, gyroscopic, and compass information along the x-axis (forward/backwards motion) y axis (left/right motion) and z axis (up/down motion) (AppleInc 2021). 8) The distance between the reference point and participant response (i.e., the end error) was recorded and used as a proxy measure of path integration, similar to previous studies [20, 21].

**Table 1:** The vestibular rotation task rotations.

Trial	Degree of movement
1	90
2	90 ⇒ -210
3	120
4	300
5	145 ⇒ -230
6	230 ⇒ -130
7	-225 ⇒ 290⇒60
8	-290 ⇒ 265 ⇒-70
9	-200 ⇒ 310 ⇒-190

Degree of movement represents the rotations in each trial. Positive and negative values represent clockwise and anticlockwise rotations, respectively. Multiple rotations are separated by ⇒ symbol (i.e., multiple rotations before the participant points back to the starting point).

### Vestibular features

The sensor data that was derived from accelerometer, gyroscope, and compass underwent pre-processing steps that are detailed in the S1 File. Following the data preparation phase, feature selection was used to generate latent vestibular features from the movement data, which in turn served as proxy measures of primary vestibular graviceptors [25]. This process was guided by theoretical knowledge on path integration, the vestibular system and cognitive impairment [24, 26–29]. Vestibular features are listed in Table 2.

**Table 2:** Features.

Features (metric of measurement)	Feature description
*Path integration*	
End Error (°)	This is the only feature not derived from motion and measures basic PI performance. Scores are calculated by subtracting the final response position (by the participant) from the correct position, similar todrop error traditionally used in navigation tests of PI(termed drop error in Kunz et al., 2015a).
Total Angular Displacement (°)	Total angular displacement is the sum of all changes in compass points. For example, if a participant begins at 90 degrees and returns to zero, the perfect movement will have a total angular displacement of 90. If participants takes a longer route in their motion response, or turns the iPad backward and forward, then the displacement will increase similar to end error measured during PI.
Tilt i.e. Largest Gyroscopic Value (°)	Tilt calculates the largest (greatest magnitude +/-) gyroscopic value for each trial. Tilt orientation is a function of the vestibular system that modulates PI performance [43]. This feature represents change of tilt orientation during movement response. Each gyroscope data point was taken every 0.003 seconds.
Quick vs longer Tilt i.e Gyroscopic Rate of Change (°/s)	Average rate of change of tilt on the gyroscope data was calculated for each axis. For the rate of change, time intervals of 0.1, 0.5 and 1.0 seconds were used. For each time, intervals add all raw gyroscope data and divide it by the number of samples in the time interval. This decreases the amount of noise that might occur within the small refresh rates of the gyroscope by averaging.
Acceleration (m/s^2^)	The average acceleration feature represents the average of the accelerometer data during response movement, similar to time taken (i.e., duration) to complete PI during self-motion on virtual PI (similar to wayinfing duration; see Coughlan et al., 2019).
Jerk (m/s^3^)	Average jerk is the time derivative of acceleration [44]. For each axis, a set amount of raw acceleration in a time (0.1, 0.5, 1.0) was summed, and the difference between each interval was calculated to generate the jerk value.
Hesitations	Average hesitation represented the accumulative number of stop and start movements in any one trial. This was calculated by using the moving window transformation of raw accelerometer data (peak detection function) to remove the noise in the raw data.

The first feature represented basic path integration performance. Six movement-based vestibular features were identified. x-axis represents forward/backwards movement; y axis responses left/right movement; z axis represents up/down movement. PI: path integration.

### Statistical analysis

Simple two-tailed t-tests were used to test the significance of any demographic or neuropsychological differences between the genetic groups (ε3ε3 and ε3ε4 carriers).

### Machine learning algorithms

To determine whether vestibular features could distinguish between the genetic groups, classification models were created for each task trial. Each trial involves a different rotational movement, and it is not possible to combine all the trials. Classification accuracy of three machine learning algorithms, including Random Forest (RF) [30], Support Vector Machines (SVM) [31] and Multi-Layer Perceptron (MLP) [32] were computed. Based on the novelty of the task our choice of model variants was based on standard models that have shown to be well generalized. In the case of RF, we applied the generic approach based on [33]. Similarly, we used a standard SVM approach and examined linear and radial basis function (RBF) kernels. Linear was used as a baseline approach and radial basis function was used to capture the potential complex variation in movement features. Finally, MLP was applied as a classical Neural Network approach to classification. Using a grid search, we evaluated performance with different model parameters. To moderate against over fitting we also applied a grid search over regularization parameter represented as C in the SVM model and α in MLP model. C parameter within the SVM provides the degree of flexibility a decision boundary can have while classifying training points. Low C identifies large margins separating groups. A higher C ensures more points are classified correctly with more complex boundaries. We evaluated performance over C values of 0.5, 1, 3, 5, 10 and 20. Similarly, α combats overfitting by restricting the complexity of decision boundaries. A lower α creates a stricter boundary and searches over α values of 0.0001, 0.0005, 0.001 and 0.002.This array of standard models is used in this first experimentation to minimise assumptions and to understand which basic method is most effective in each consecutive trial. Classification is based on a standard class metric, the F1 score. The F1 score is a measure of the model’s performance that considers precision (true positives/true positives + false positives) and recall (true positives/true positives + false negatives):

$$F1=2*\frac{precision*recall}{precision+recall}=\frac{True\;positives}{True\;positives+0.5*(False\;positives+false\;negatives)}$$

**Fig 2:**
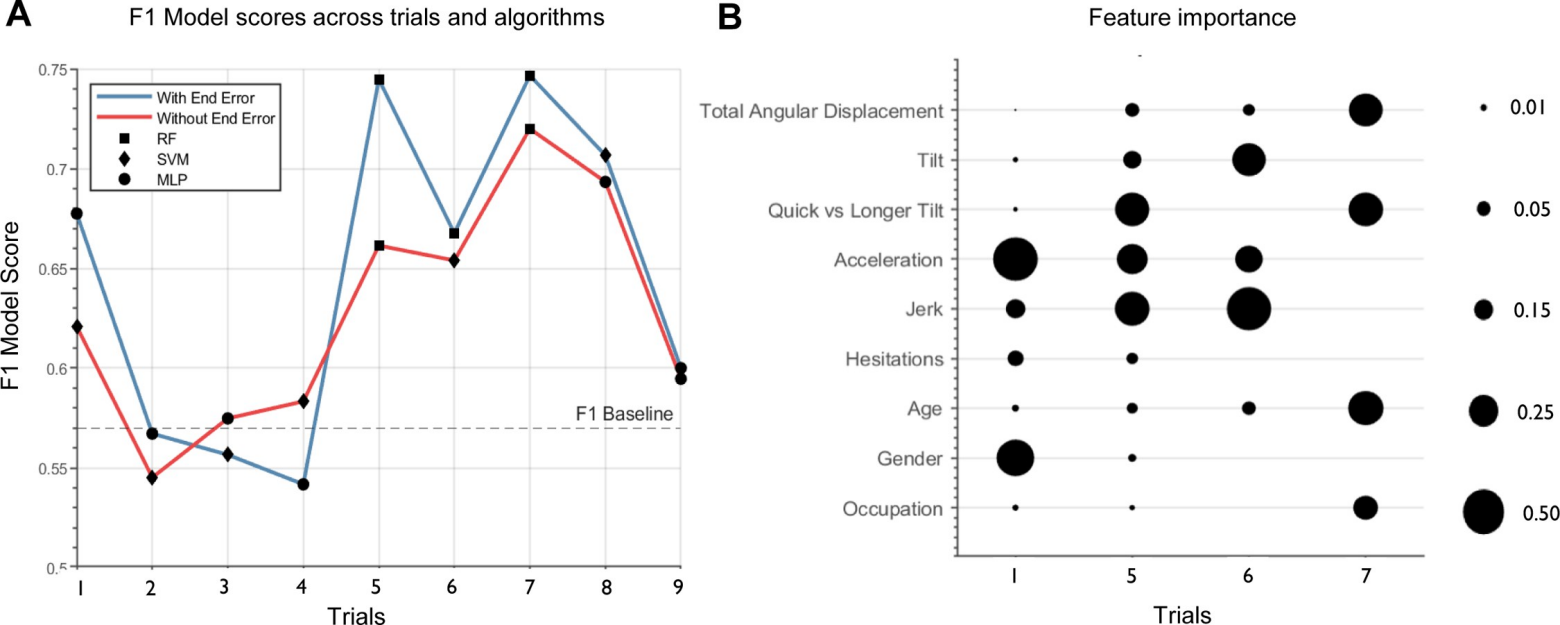
Machine learning models and feature importance. **A** F1 scores for the best performing algorithm are shown. A random predictor would score 0.57, with a score of above 0.57 representing better-than-chance *APOE* status classification performance. Blue line includes all features. Red line excludes the path integration feature, end error. **B** Importance scores are represented by the circle diameter and were derived for the best performing model on each of the trials shown. Scores vary between 0 and 1 depending on the proportion of influence the feature has for that trial. RF = Random Forest, SVM = Support Vector Machine, MLP = Multi-Layer Perception.

Thus, the F1 score determined the classification performance for *APOE* status (i.e., ε3ε3 vs ε3ε4). A random classification F1 score is equal to 0.57, with a score of above 0.5 suggesting improved efficacy/sensitivity. Accuracy scores were also calculated as a secondary metric. Accuracy represents the percentage of correctly classified ε3ε3 and ε3ε4 participants. Two sets of analyses were performed: in the first set of models, all features were included (blue line in Fig 2A). In the second set of models, end error was excluded to assess the independent precision of the movement-based features to the prediction of genetic risk (red line in Fig 2A). An overview of the machine learning algorithms is provided in S1 File.

### Cross-validation

A control validation resampling procedure evaluated the machine learning models [34]. Five-fold validation was used using four groups as training data and one group for testing. We selected these splits such that each participant was included in either the training or test dataset. Five F1 scores for each algorithm and for each trial were computed and averaged. Each of five groups are created using stratified folds to preserve the samples in both *APOE* groups.

## Results

### Demographic characteristic

Demographic and neuropsychology characteristics were not significantly different between genetic groups (Table 3). Secondary characteristics are presented in S1 Table in S1 File.

**Table 3:** Primary demographic and neuropsychology characteristics of the sample.

Measure	ε3ε3 (n = 32)	ε3ε4 (n = 21)	*P* value (F)
Age (years)	63.63 (5.79)	62.95 (5.53)	
Mean (SD)			-
Sex			
*Male*	17	6	-
*Female*	15	15	-
ACE-III	94.10 (4.64)	94.67 (2.43)	.85 (0.04)
*Attention*	17.30 (1.01)	17.71 (0.43)	.13 (.236)
*Memory*	25.17 (1.08)	25.01 (1.09)	.72 (.392)
*Fluency*	12.30 (2.01)	12.43 (1.05)	.89 (.235)
*Language*	25.07 (1.23)	25.29 (0.83)	.63 (.233)
*Visuospatial*	14.27 (1.33)	14.24 (0.91)	.71 (.264)
ROCT			
*Recall*	21.92 (5.5)	21.66 (5.24)	.87 (.223)
*Copy*	33.77 (2.67)	32.12 (2.37)	.27 (1.287)

Primary demographic and neuropsychological characteristics of the genetic groups (Independent sample t-test, two-tailed). ACE-III = Addenbrooke’s Cognitive Examination III, ROCT = The Rey–Osterrieth complex Fig task- recall administered three minutes after copy.

### Vestibular features distinguish ε4 carriers from non-carrier controls

Next, we sought to examine if the vestibular features distinguished the genetic groups by applying a machine learning approach. The three chosen algorithms examined the classification performance of the vestibular features for *APOE* genotype status. The vestibular features are listed in Table 2. Six out of the nine trials achieved a cross-fold average F1 score of above 0.6 using one or more of the algorithms, suggesting stable differences in vestibular function among *APOE* ε3ε3 and ε3ε4 carriers. Accuracy percentages ranged from 65% to 79% depending on task trial and algorithm. Excluding age, sex and occupation data produced similar F1 scores, suggesting that vestibular function classified *APOE* status irrespective of demographic variation (see S2 Table in S1 File). Please refer to S3 Table in S1 File for results including a small number of highest-genetic-risk ε4ε4 carriers who were included in the *APOE* ε4 carrier group, subsequently producing greater F1 scores (reaching 0.77) across all trials. We then examined the influence of the vestibular function when the path integration proxy ‘end error’ was excluded. Excluding this feature led to a best prediction accuracy of 0.75, suggesting that the movement-based features alone maintained good classification performance for *APOE* status. Accuracy ranged from 65%-75%. The highest performing algorithm for each trial including and excluding path integration is presented in Fig 2A. F1 scores and accuracy scores across all trials and the best performing algorithm are presented in Table 4.

**Table 4:** Machine learning F1 and accuracy scores.

**A. Including end error**				
Trial	F1 Score	Algorithm(F1)	Accuracy Score	Algorithm (Accuracy)
1	0.677	MLP	0.752	RF
2	0.567	MLP	0.655	MLP
3	0.557	SVM	0.696	MLP
4	0.542	MLP	0.663	SVM
5	0.745	RF	0.790	RF
6	0.669	RF	0.750	RF
7	0.747	RF	0.720	RF
8	0.707	SVM	0.670	MLP
9	0.600	MLP	0.683	MLP
**B. Excluding end error**				
Trial	F1 Score	Algorithm(F1)	Accuracy Score	Algorithm (Accuracy)
1	0.621	SVM	0.700	MLP
2	0.545	SVM	0.645	MLP
3	0.575	MLP	0.653	MLP
4	0.583	SVM	0.696	SVM
5	0.661	RF	0.745	RF
6	0.654	SVM	0.736	RF
7	0.720	RF	0.670	RF/SVM
8	0.693	MLP	0.710	MLP
9	0.595	MLP	0.690	MLP

**A** All features including end error, demographics (age, sex and occupation and all vestibular features. **B** The non- motion-based path integration feature; end error was excluded. F1 scores reflect the best performing models and algorithm on each trial. A random predictor would score 0.57 or lower, with a score of above 0.57 representing task prediction efficacy. Accuracy scores reflect the percentage of correctly classified ε3ε4 and ε3ε3.RA = Random Forest, SVM = Support Vector Machine, MLP = Multi-Layer Perceptron.

### Identifying the most influential vestibular features

Features with the best classification precision and accuracy varied across task trials (Fig 2B). Demographics are included as a means of comparison.

## Discussion

Our results show that a novel movement-based vestibular task can classify vestibular changes in at-genetic-risk of AD, irrespective of demographic background. Machine learning algorithms achieved good performance (F1 and accuracy scores of up to 0.72 and 0.75, respectively) based on the derived vestibular features of the task. We also replicated previous path integration deficits in *APOE* ε4 gene carriers when task novelty was high [20, 24]. Our findings highlight the need for a broader computational perspective to understand the at-genetic-risk AD phenotype.

In more detail, we developed the Vestibular Rotation Task and applied a feature selection approach to reduce the compass, accelerometer and gyroscope data into meaningful vestibular features that characterise the human vestibular system, as well as basic path integration performance. The features engineered included: ‘end error‘ (the basic path integration performance measure), angular displacement, total tilt, change in tilt, acceleration, hesitations, and average jerk. Three machine algorithms tested the classification accuracy of the features to detect *APOE* ε4 carriership status, achieving a prediction accuracy of up to 75%. After removing the PI accuracy measure, ‘end error’, the best prediction accuracy reduced by just 3% (to 72% accuracy), suggesting that the motion features distinguished *APOE* ε4 carriers from non-carriers.

### Dysregulation of the vestibular system is associated with path integration deficits

Evidence from animal models has consistently demonstrated the importance of the vestibular system to path integration. Vestibular lesions impair spatial memory and the ability to rerun to a goal location following passive transport [35]. This effect is exacerbated in darkness [36]. Interestingly, rodents with vestibular lesions path integrate successfully with the aid of external visual cues [37]. This has striking similarities to Bierbrauer and colleagues who found that a path integration deficit in *APOE* ε4 carriers emerges only if external visual cues are not available [20]. These findings are also consistent with limited studies that show path integration impairment in vestibular deficient humans emerges when external cues are not available [38], supporting the emerging theory that vestibular function plays a mechanistic role in path integration deficits previously observed in adults at-genetic-risk AD.

### Clinical implications

Vestibular signals that influence path integration in preclinical AD may help pinpoint pre-disease pathological changes and in turn guide treatment. Identifying vestibular contributions to the cognitive phenotype of preclinical AD is important because vestibular dysfunction is often present with treatable hearing loss [39], recently cited as a modifiable risk factor for AD [40]. Moreover, vestibular balance training such as Intensive Slackline-Training improves path integration and vestibular function and implanted vestibular prosthesis (that reproduces information normally provided by semi-circular canals) improves spatial orientation in monkeys with severe vestibular damage [33], suggesting adults with vestibular dysfunction (and path integration impairments), may respond to a vestibular implant and or vestibular intensive training. Moreover, because the vestibular system has extensive connections to AD vulnerable brain regions including the hippocampus, cingulate cortex and parietal lobe, vestibular stimulation may indeed improve cognitive performance related to the integrity of these brain regions, including disorientation and memory loss. Our approach to investigating vestibular contributions should be considered a steppingstone towards more tailored treatment programs for preclinical AD, that can be combined with pharmacological, non-pharmacological sensory stimulation, gamma-induction, and dietary treatment strategies [41, 42].

### Limitations, future research directions and conclusion

Vestibular contributions to the cognitive phenotype of at-genetic-risk AD warrant further investigation. Our study included an *APOE* genotyped sample that was a similar sample size to previous studies [21, 24]. Nonetheless, this sample is moderate in the context of machine learning approaches. A further challenge when applying supervised machine learning methodology is the correct creation and selection of movement features. As more research is conducted into the vestibular system in the context of cognitive impairment, additional movement-based features can be created and used as predictors. To prevent the high dimensionality of features, a naïve correlation feature selection was applied to reduce the number of input dimensions. Moreover, signals from the device can contain noise and unwanted artifacts. We implemented a bespoke code to pre-process to counteract this. Simple heuristics applied to the raw signal included flipping movement when a participant held the iPad rotated 180° and applying a threshold of 80° for sequential compass values. To extract potential hesitations, the raw data was filtered by a moving window averaged over 100 data points and smoothing the raw signal such that we could implement a peak detection for hesitations. From a theoretical perspective, further research is needed to understand how degraded graviception contributes to deficits in self-motion perception and the role played by head direction and grid cell dysfunction in graviception.

In conclusion, we introduce a novel movement-based task of human vestibular function and path integration. The application of machine learning revealed movement-based vestibular changes in at-genetic-risk adults, which should be a priority for basic scientific research in AD. Our findings may accelerate objective, high frequency and passive digital phenotyping of at-genetic-risk AD and help elucidate the mechanisms by which the human vestibular system contributes to cognitive impairment in preclinical AD. Future studies may examine whether grid cell function, detectable in task-based fMRI, is causally linked to vestibular dysfunction in at-genetic-risk AD and secondly, whether vestibular changes precede, or succeed, the emergence of tauopathy in the brainstem and entorhinal cortex during the preclinical stage of AD.

## Supporting information

S1 File (DOCX)

## Abbreviations

PI: Path Integration
APOE: Apolipoprotein E
RF: Random Forest
SVM: Support Vector Machine
MLP: Multi-Layer Perception
