# Supplementary material for: Vestibular contribution to path integration deficits in ‘at-genetic-risk’ for Alzheimer’s disease
Source: PLoS One. 2023 Jan 3;18(1):e0278239. doi: 10.1371/journal.pone.0278239 (PMC9810179; doi:10.1371/journal.pone.0278239)
Supplement: S1 File — (DOCX) [file pone.0278239.s001.docx]

**Supplementary Materials for**

**Vestibular deficits in ‘at-genetic-risk’ for Alzheimer’s disease**

**Authors: Gillian Coughlan, William Plumb, Peter Zhukovsky, Min Hane Aung, Michael**

**Hornberger**

**Correspondence to:** [**m.hornberger@uea.ac.uk**](mailto:m.hornberger@uea.ac.uk)

**This PDF file includes:**

**Materials and Methods**

**Figs. S1 to S2**

**Tables S1 to S3**

Materials and Method

**Pre-processing preceding feature extraction**

Python (version 3.2) and R studio (version 1.4) were used for pre-processing and statistical analysis. A signal pre-processing stage was first applied to the raw accelerometer, gyroscope and compass sensor data in order to mitigate signal noise and instrumental artefacts. This in turn allowed us produce signals that represented accurate response movements, which were given by the participant at the end of each trial. Noise removal involved using moving window averaging filters to smooth by averaging over 0.1 seconds. We used a set of cleaning rules (also known as bespoke heuristics) to correct for sensor errors such as unnatural movements. For each participant, a different reference/start point was set on the iPad prior to the task. To enable analysis between each participant the starting orientation was set to have the same compass value. Subtracting each participants’ reference start point from their current location produced the start angle. If instantaneous changes in the raw signals occurred due to hardware-based errors in the raw compass data, sensor errors, or measurement errors, the implementation applied simple addition or subtraction at each change point. This removed large value changes caused by the hardware set at changes of over 80 degrees between sequential data points. This threshold was chosen as it near impossible that a participant would turn the iPad 80 degrees within 0.1 seconds and thus such changes must be a result of sensor artefacts. If the participants held the iPad reversed hence the movement was inverted, the values were mirrored so all movements were in the same direction. These steps ensured that all the heading data was comparable between participants and thus we calculated the orientation difference between the start points and current compass positions, which is comparable between the participants. A raw and processed data example is represented in Fig. S2. Following data processing, the participant numbers for each group (ε3ε3, ε3ε4) on each trial include: Trial 1 (32,21), Trial 2 (32,21) Trial 3 (31,21) Trial 4 (32,21) Trial 5 (19,16) Trial 6 (32,21) Trial 7 (11,10) Trial 8 (11,10) Trial 9 (31,21).

**Feature collinearity**

Following feature selection ^8^, Spearman’s correlations were used to assess multicollinearity between the vestibular features generated in Table 1 (see Fig. S1 for a heat map representation the correlation between features [x, y, and z axis of the iPad summed] on trial one). For the Spearman’s rank correlations for each pair of features, a correlation threshold was set to reduce multicollinearity. The method was initiated by starting with “end error” and evaluating all correlation values against it. If the correlation with a further feature does not exceed the threshold, then it is added to a subset of new subset of features with “end error”. Using the next feature in the subset, we check each pair of features and remove one that correlates over the threshold. This continued until all features pairs in the subset were evaluated (i.e. until all pairs of features in the subset are less correlated than the threshold value). We test this with a set of threshold values {spearman’s r: 0.05, 0.1, 0.2, 0.3, 0.5, 1.0} to gain multiple feature sets allowing us to evaluate the best feature set for the machine learning algorithms, with 0.05 being the most stringent threshold (no features in the prediction model) and 1.0 is the most relaxed threshold (all features in the model).

**Fig. S1 Correlation heat map for trial one using Spearman's rank correlation.**

The correlation value was calculated for each pair of features to set a correlation threshold to remove any feature that correlates highly with other features. The correlation threshold was varied from 0.05, 0.1, 0.2, 0.3, 0.5, 1. The method is initiated by adding the first feature (end error) and then checking all correlation values against it. If any other feature breach this threshold, it is removed from the original list of features. The next feature in the original feature list is then selected until none remain.

**
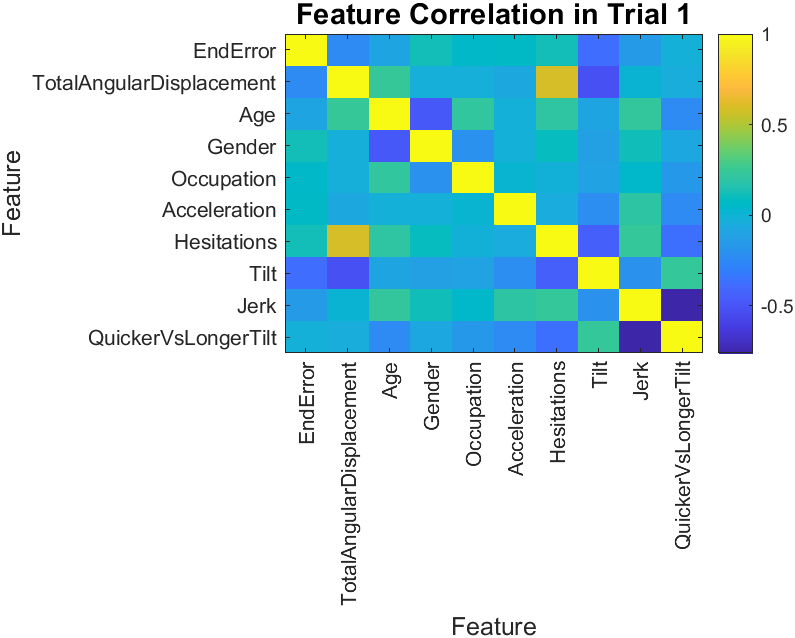
**

**Fig. S2*.* Example of signal pre-processing which allowed us to clean heading orientation from the raw compass signal (left) to the clean compass signal (right).**


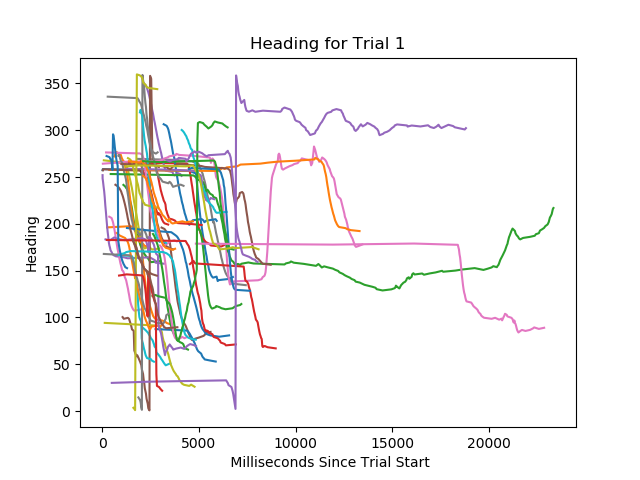

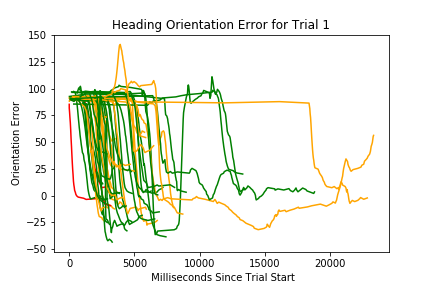


### Machine learning algorithms

*The Random Forest Algorithm* (RF) comprises of decision trees and was chosen due to its applicability to large features sets via as pruning. A set of features is selected to train a decision tree based on the *APOE* genotype. The maximum number of features per tree and number of trees was set. Each individual tree selects features that best separate the *APOE* target, meaning less important features will not be used. The final model is used to benchmark any prediction results against, as it includes all features. Two variable parameters for the RF model were tested, the first being the number of decision trees used in the ensemble, where 100, 250, 500 and 1000 trees were tested. The second being the maximum number of features available to each tree. We applied the proportional factors 0.2, 0.5 and 0.7 which were multiplied by the total number of features.

*Support Vector Machine* (SVM) is a widely used type of classification model based on the optimization of an *N*-dimensional hyperplane, where *N* is the number of features. Linear and non-linear radial kernels were used. Values of the parameter *C* which controls the margin of the hyperplane was tested with the values (0.5, 1, 3, 5, 10 and 20).

*Multi-Layer Perceptron* (MLP) is a widely used feed forward neural network that can be applied to classification tasks. For the MLP, we tested three types of the activation functions of the neurons (identity function, logistic function and the hyperbolic function), the alpha constant, which is a parameter used to constrain the flexibility of the model to prevent overfitting {0.0001, 0.0005, 0.001, 0.002} and the number of nodes in the hidden layer {5, 10, 15, 20}.

**Table S1. Secondary characteristics of the sample***.* Secondary characteristics between genetic groups. No difference on any of the above listed characteristics were detected with Persons Chi square confirmed analysis.

| Measure |  |  | ε3ε3 | ε3ε4 | χ2 (df) | p value |
| --- | --- | --- | --- | --- | --- | --- |
| Marital Status |  |  |  |  |  |  |
|  | Single |  | 10 | 3 | 2.687 (2) | *ns* |
|  | Partner/married |  | 22 | 18 |  |  |
| Education |  |  |  |  |  |  |
| ^(missing N=1)^ | >=8years |  | 5 | 2 | 1.977 (4) | *ns* |
|  | =11years |  | 5 | 5 |  |  |
|  | =14years |  | 8 | 4 |  |  |
|  | =15years |  | 14 | 9 |  |  |
| Blood pressure |  |  |  |  |  |  |
| ^(missing N=1)^ | Not medicated |  | 25 | 16 | .026 (1) | *ns* |
|  | Medicated |  | 7 | 4 |  |  |
|  |  |  |  |  |  |  |
| Cholesterol |  |  |  |  |  |  |
|  | Not medicated |  | 26 | 19 | 1.997 (1) | *ns* |
|  | Medicated |  | 6 | 1 |  |  |
|  |  |  |  |  |  |  |
| Family History of AD |  |  |  |  |  |  |
| ^(missing n=4)^ | None |  | 21 | 8 | 2.066 (2) | *ns* |
|  | One parent |  | 7 | 7 |  |  |
|  | Both parents |  | 4 | 2 |  |  |
| Occupation |  |  |  |  | 6.494 (3) | *ns* |
| ^(missing n=2)^ | Manual/Unskilled |  | 5 | 7 |  |  |
|  | Skilled |  | 9 | 13 |  |  |
|  | Professional |  | 17 | 10 |  |  |

**Table S2.** Model performance excluding demographic variables (age, sex and occupation)

| Trial | Best F1 Score | Algorithm |
| --- | --- | --- |
| 1 | 0.630 | RF |
| 2 | 0.568 | MLP |
| 3 | 0.596 | MLP |
| 4 | 0.520 | SVM |
| 5 | 0.745 | RF |
| 6 | 0.688 | MLP |
| 7 | 0.733 | MLP |
| 8 | 0.720 | MLP |
| 9 | 0.626 | MLP |

**Table S3.** F1 scores reflecting for the best performing prediction models and algorithm on each trial, with ε4ε4 participants included (N=3).

| Trial | Best F1 Score | | Algorithm |
| --- | --- | --- | --- |
| 1 | 0.673 | MLP | |
| 2 | 0.582 | MLP | |
| 3 | 0.587 | MLP | |
| 4 | 0.599 | MLP | |
| 5 | 0.767 | RF | |
| 6 | 0.705 | RF | |
| 7 | 0.765 | RF | |
| 8 | 0.734 | MLP | |
| 9 | 0.703 | MLP | |

**Discussion**

This study uses five-fold cross validation to evaluate the models. In our cross validation, we select splits such that each participant’s data is only included either training or test datasets but not both. This is to ensure models are not fitting to data from a specific person whose data would also be included in the test dataset. To moderate against over fitting we also applied a grid search over regularization parameter represented as C in the SVM model and a in MLP model. C parameter within the SVM provides how much leeway a decision boundary can have while classifying training points. Low C looks for large margins separating groups, higher C ensures more points are classified correctly with more complex boundaries. We evaluate performance over C values of 0.5, 1, 3, 5, 10 and 20. Similarly, a combats overfitting by restricting the complexity of decision boundaries. Lower a creates a stricter boundary. Searching over varying a of 0.0001, 0.0005, 0.001 and 0.002. A further challenge when applying supervised machine learning methodology is the correct creation and selection of movement features. As more research is conducted into the vestibular system and cognitive impairment, improved feature can be created and used as predictors. As to prevent high dimensionality of features, we also applied a naïve correlation feature selection to reduce the number of input dimensions. Moreover, signals from a tablet can contain noise and unwanted artifacts in the data. We implement a bespoke code to pre-process this information. Simple heuristics applied to the raw signal include, flipping movement when a participant holds the iPad rotated 180° and applying a threshold of 80° for sequential compass values in which we remove this unrealistic movement to the previous value. To extract potential hesitations, the raw data is filtered through moving window averaging over 100 data points, smoothing the raw signal such that we can implement peak detection for counts of hesitations.

**References**

1. Mittelstaedt ML, Mittelstaedt H. Homing by path integration in a mammal. *Naturwissenschaften*. 1980;67(11):566–567.

2. Mittelstaedt ML, Mittelstaedt H. Idiothetic navigation in humans: Estimation of path length. *Experimental Brain Research*. 2001;139(3):318-332. doi:10.1007/s002210100735

3. Mittelstaedt H, Mittelstaedt ML. Homing by Path Integration. In: Springer, Berlin, Heidelberg; 1982:290-297. doi:10.1007/978-3-642-68616-0_29

4. Mittelstaedt H. The role of the otoliths in perception of the vertical and in path integration. *Annals of the New York Academy of Sciences*. 1999;871:334-344. doi:10.1111/j.1749-6632.1999.tb09196.x

5. Cheng Z, Gu Y. Vestibular system and self-motion. *Frontiers in Cellular Neuroscience*. 2018;12:456. doi:10.3389/fncel.2018.00456

6. Angelaki DE, Klier EM, Snyder LH. A Vestibular Sensation: Probabilistic Approaches to Spatial Perception. *Neuron*. 2009;64(4):448-461. doi:10.1016/j.neuron.2009.11.010

7. Chen X, DeAngelis GC, Angelaki DE. Diverse spatial reference frames of vestibular signals in parietal cortex. *Neuron*. 2013;80(5):1310-1321. doi:10.1016/j.neuron.2013.09.006

8. Hall MA. Correlation-based Feature Selection for Machine Learning. 1999;(April).
